# Synthesis of a tyrosinase inhibitor by consecutive ethenolysis and cross-metathesis of crude cashew nutshell liquid

**DOI:** 10.3762/bjoc.14.252

**Published:** 2018-10-31

**Authors:** Jacqueline Pollini, Valentina Bragoni, Lukas J Gooßen

**Affiliations:** 1 Lehrstuhl für Organische Chemie I, Ruhr-Universität Bochum, ZEMOS, Universitätsstraße 150, 44801 Bochum, Germany

**Keywords:** cashew nutshell liquid, cross-metathesis, renewable feedstock, sustainable chemistry, tyrosinase inhibitor

## Abstract

A convenient and sustainable three-step synthesis of the tyrosinase inhibitor 2-hydroxy-6-tridecylbenzoic acid was developed that starts directly from the anacardic acid component of natural cashew nutshell liquid (CNSL). Natural CNSL contains 60–70% of anacardic acid as a mixture of several double bond isomers. The anacardic acid component was converted into a uniform starting material by ethenolysis of the entire mixture and subsequent selective precipitation of 6-(ω-nonenyl)salicylic acid from cold pentane. The olefinic side chain of this intermediate was elongated by its cross-metathesis with 1-hexene using a first generation Hoveyda–Grubbs catalyst, which was reused as precatalyst in a subsequent hydrogenation step. Overall, the target compound was obtained in an overall yield of 61% based on the unsaturated anacardic acid content and 34% based on the crude CNSL.

## Introduction

**Scheme 1:**
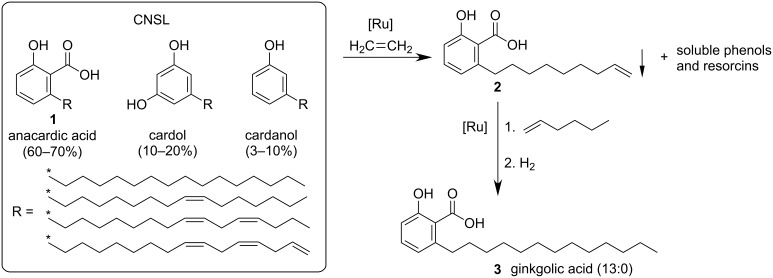
Targeted conversion of CNSL into a tyrosinase inhibitor.

Cashew nutshell liquid (Scheme 1) is an ideal renewable feedstock. This non-edible industrial waste product, derived from the cashew nut processing, is abundant available and cheap [1–3]. The annual production of cashew nuts with shell reached 4.9 million tons in 2016 [4], leading to an estimated CNSL production of 1.2 million tons per year [5]. CNSL is a mixture of phenolic compounds such as anacardic acid (**1**), cardol and cardanol, each bearing a C-15 side chain in *meta*-position to the hydroxy group with a varying degree of unsaturation [6].

CNSL exhibits a broad range of biological properties and industrial applications, for instance in surfactants, plasticizers, resins, soft materials and diverse medical applications [7]. Isolated via cold-press or solvent extraction processes, it contains predominantly anacardic acid (**1**). Upon distillation or any other thermal treatment, anacardic acid is known to decarboxylate easily with formation of technical cashew nutshell liquid (tCNSL), which consists mainly of cardanol. Due to this industrial processing method, the main focus in research aiming at the chemical valorization and modification of CNSL is on cardanol-derived products [8–10]. These include aromatic amines as polymers [11–12], cardanol-based phosphates as modifiers for epoxy resins [13], cardanol grafted natural rubber as rubber plasticizers [14], amine-based surfactants [15] and phenol/cardanol-formaldehyde based adhesives [16].

The chemical valorization of anacardic acid (**1**) is even more attractive, because it contains an additional functional group. However, the separation and purification of this CNSL component without decarboxylation is laborious and relies on wasteful and tedious processes such as fractionate precipitation or column chromatography [6,17]. A limited number of derivatizations of anacardic acid are reported by now, including the synthesis of lactones [18–20], sulfonamides [21] or hydrazones [22], typically bioactive compounds though with low commercial value. However, several studies suggest that anacardic acid and its derivatives display a broad range of biological activities such as antimicrobial [23], antioxidant [24], molluscicidal [25] and antiplaque [26]. Ginkgolic acids, structurally closely related analogues of anacardic acid, have been reported to exhibit tyrosinase inhibitory activity [27]. We herein report a concise synthesis of the most potent tyrosinase inhibitor among them, the ginkgolic acid (13:0), starting from crude CNSL (Scheme 1, left).

Tyrosinase is an enzyme [28] which is responsible for browning of fruits and vegetables as well as skin pigmentation [29]. Furthermore, it is linked to several neurodegenerative diseases [30]. Therefore, the study and development of tyrosinase inhibitors from renewable resources is of particular interest for research and industry [31–32]. Fu et al. investigated naturally occurring ginkgolic acids which they selectively synthesized from 2,6-dihydroxybenzoic acid (**4**), and found that the tridecanyl substituted derivative ginkgolic acid (13:0, **3**) exhibits the most promising inhibitory activity.

**Scheme 2:**
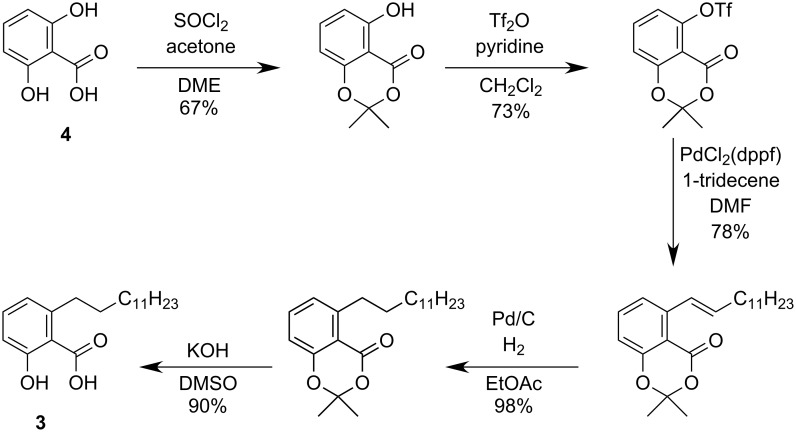
Previous synthesis of 2-hydroxy-6-tridecylbenzoic acid by Fu et al.

While this modular approach is very appealing for drug-discovery, the use of expensive γ-resorcylic acid as the substrate basis and the low overall yield over several reaction steps are certainly drawbacks for larger scale production (Scheme 2) [27].

Due to the structural similarity of ginkgolic and anacardic acids, we believed that a particularly desirable synthesis of 2-hydroxy-6-tridecylbenzoic acid (**3**) would involve CNSL as the substrate basis. However, the functionalization of the anacardic acid component of CNSL presents several challenges. Since CNSL consists of a mixture of acids, phenols and resorcins with saturated and unsaturated side chains, it seemed to be impossible to derive a single product with a shorter side chain via a cross-metathesis with a short olefin, since inevitable, an inseparable mixture of many compounds would result. It is, thus, necessary to converge as many components as possible into one single compound.

Based on concepts that we had previously utilized for the chemical modification of the cardanol component, we first investigated strategies based on cross-metathesis of CNSL with ethylene [33–35]. Each unsaturated double bond isomer has the first double bond located at the C-8 position, so that no matter how many other double-bonds are present, the unsaturated side chains of all arenes will be shortened to ω-nonenyl groups if ethylene is added in excess. The main difficulty is that thermal purification of CNSL would inevitably lead to decarboxylation, and that unpurified CNSL, as it is obtained in an extraction process, contains a wealth of side components, many of which act as catalyst poisons.

However, if an ethenolysis could be conducted with crude CNSL, it would lead to the shortened derivatives of all unsaturated components. We reasoned that it might be possible to selectively precipitate the 2-hydroxy-6-(non-8-enyl)benzoic acid (**2**) from this product mixture and use this as a substrate for a consecutive cross-metathesis with 1-hexene followed by a hydrogenation and thus, selectively obtain the target product 2-hydroxy-6-tridecylbenzoic acid (**3**).

## Results and Discussion

### Ethenolysis of crude CNSL

**Scheme 3:**
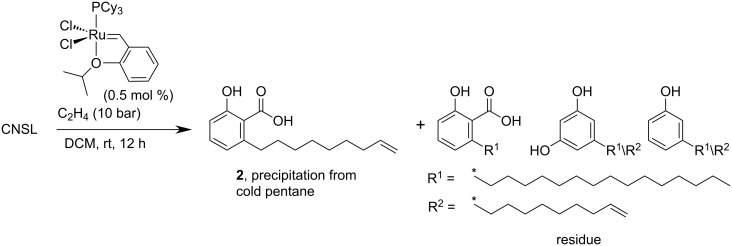
Ethenolysis of the crude CNSL.

After thorough optimization, we found that natural CNSL, a highly viscous brown oil, obtained by ether extraction of cashew nutshells, undergoes smooth ethenolysis only in dichloromethane as the solvent (Scheme 3). Using more sustainable solvents or no solvent at all, the reaction gave almost no turnover, regardless of the ruthenium catalyst employed. However, as a 1.1 M solution in dichloromethane, the unsaturated components of CNSL were converted in high yields at 10 bar of ethylene in the presence of 0.5 mol % of the first generation Hoveyda–Grubbs catalyst **Ru-1**.

The resulting mixture was filtered through celite, and the dichloromethane solvent was removed in vacuo. After addition of pentane, the mixture was chilled causing selective precipitation of the desired product **2** as a colorless solid in an amount that is equivalent to 80% of the anacardic acid content or 84% of the unsaturated anacardic acid. Anacardic acid makes up for ca. 70% of the CNSL, so that the yield is 56% based on the entire CNSL. We were pleased to find that the saturated C15-anacardic acid stays in solution along with cardanol and cardol derivatives. This residue may be utilized for chemical valorization after purification via distillation. This ethenolysis/purification sequence was successfully performed on multi-gram scales, yielding up to 16 g product in a single run.

### One-pot cross-metathesis/hydrogenation

We next sought for suitable conditions that would allow the cross-metathesis of **2** with 1-hexene to give 2-hydroxy-6-(tridec-8-enyl)benzoic acid (**5**). When performing the hexenolysis of **2** with 7 equivalents of 1-hexene using 1 mol % of **Ru-1** in dichloromethane at rt, the desired product was obtained only in unsatisfactory yield after 12h (Table 1, entry 1). High amounts of starting material were detected in the reaction mixture which points towards either a low conversion or an unfavorable position of the metathesis reaction equilibrium. We tested several methods to shift the equilibrium by purging the ethylene byproduct from the reaction mixture with inert gas, but finally found that the best yields were obtained when allowing the ethylene to slowly evaporate from the reaction mixture via an oil bubbler. This way, the yield was improved to 53% (Table 1, entry 2).

**Table 1:** Cross-metathesis of 2-hydroxy-6-(non-8-enyl)benzoic acid (**2**) with 1-hexene.^a^

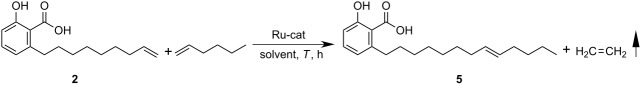						

entry	catalyst	solvent	1-hexene [equiv]	time	conversion [%]	**5** [%]^b^

1^c^	**Ru-1**	DCM	7	12 h	35	33
2^d^	**Ru-1**	DCM	7	12 h	55	53
3	**Ru-1**	DCM	7	12 h	97	73
4	**Ru-1**	*p*-cymene	7	12 h	28	3
5	**Ru-1**	DMC	7	12 h	66	44
6	**Ru-1**	Me-THF	7	12 h	64	47
7	**Ru-1**	acetone	7	12 h	76	59
8	**Ru-1**	THF	7	12 h	51	42
9	**Ru-1**	DCM	5	12 h	94	69
10	**Ru-1**	DCM	3	12 h	81	65
11	**Ru-1**	DCM	7	6 h	96	74
12	**Ru-2**	DCM	7	6 h	98	72
13	**Ru-3**	DCM	7	6 h	98	65
14	**Ru-4**	DCM	7	6 h	98	56
15	**Ru-5**	DCM	7	6 h	93	55
16	**Ru-6**	DCM	7	6 h	46	27
17	**Ru-7**	DCM	7	6 h	98	45
18^e^	**Ru-1**	DCM	7	6 h	97	76 (72)^f^

^a^Reaction conditions: 0.5 mmol **2**, given equiv 1-hexene, 1 mol % Ru-cat, 60 °C, given time, open system via oil bubbler, ^b^Yields determined by GC using *n*-tetradecane as internal standard. ^c^rt, closed system; ^d^rt; ^e^2 mol % Ru-cat; ^f^isolated yield.

The yield was further improved by raising the reaction temperature to 60 °C (Table 1, entry 3). Now, only 3% starting material **2** was detected, but unwanted homocoupling of **2** (product **6**, see Supporting Information File 1) became a major side reaction.

We tested several solvents including sustainable solvents like dimethyl carbonate and *p*-cymene. Unfortunately, this led to a decreased conversion and just 44–47% yield of the desired product. The use of the halogenated solvent dichloromethane was still most efficient. Comparative tests with varying amounts of 1-hexene revealed that an excess of 7 equivalents was optimal. With a smaller amount the yield was decreased (Table 1, entry 9 and 10), while a higher excess leads to decreased conversion. This can be explained by the undesired homocoupling of 1-hexene as a side reaction, which delivers the less active 5-decene (**7**, see Supporting Information File 1). In principle, these internal olefins can still undergo metathesis albeit with less activity, depending on the catalyst. It was possible to reduce the time of the reaction to 6 h with almost the same yield (Table 1, entry 11).

**Figure 1:**
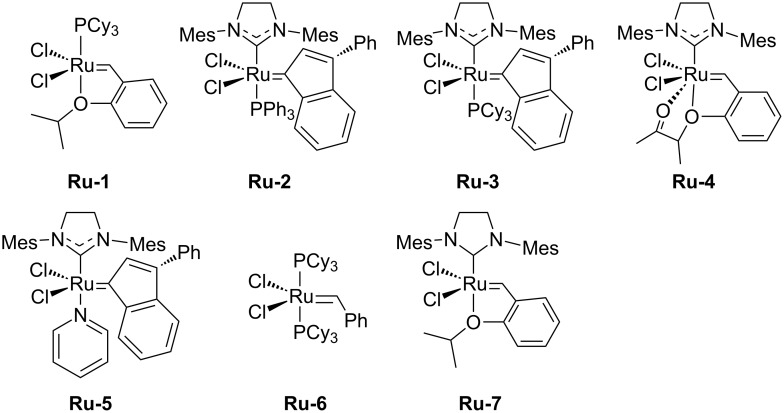
State-of-the-art metathesis catalysts.

We investigated various ruthenium catalysts in search for the optimal performance (Figure 1). The second generation Hoveyda–Grubbs catalyst previously used to change the olefinic side chain of cardanol via cross-metathesis [36], only reached a yield of 45% (Table 1, entry 17). Several modified second generation catalysts were tested, reaching yields of up to 72% of the desired product (Table 1, entry 12). However, the first generation Hoveyda–Grubbs catalyst **Ru-1**, which was reported in literature to be highly efficient for the ethenolysis of several CNSL components [35], showed the best activity. Increasing the catalyst loading to 2% gave only insignificantly better yields (Table 1, entry 18).

**Scheme 4:**
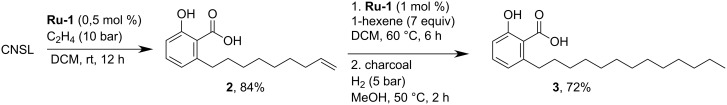
Overall process in a preparative scale.

It is known that ruthenium metathesis catalysts can be transformed in situ into an active hydrogenation catalyst [37–38]. We, thus added charcoal and methanol to the crude reaction mixture of the cross-metathesis and stirred the reaction for additional 2 h under 5 bar of hydrogen. This way, the products were fully hydrogenated in quantitative yield. We were pleased to find that the desired product **3** could easily be purified by fractionate precipitation from cold pentane. The one-pot cross-methathesis/hydrogenation was successfully scaled up to multi-gram (8 mmol) scale yielding 72% of the hydrogenated product **3**. Combined with the ethenolysis/precipitation step, the entire sequence afforded 61% overall yield based on unsaturated anacardic acids present in the CNSL (Scheme 4).

## Conclusion

In conclusion, a straightforward sequence of an ethenolysis, cross-metathesis and hydrogenation was developed for the synthesis of the tyrosinase inhibitor **3** from the non-edible waste product CNSL. The key step to this process is the ethenolysis of crude CNSL followed by a selective precipitation of 2-hydroxy-6-(non-8-enyl)benzoic acid (**2**), which transforms the complex substrate mixture into a single, pure compound. The subsequent hexenolysis can be combined with an hydrogenation to an efficient one-step process to obtain the target molecule 2-hydroxy-6-tridecylbenzoic acid (**3**). Interestingly, it is a first-generation Hoveyda–Grubbs catalyst **Ru-1** that is most efficient for both metathesis steps.

## Experimental

### General methods

All reactions were performed in oven-dried glassware containing a Teflon-coated stirring bar and dry septum under argon atmosphere. All optimization reactions were monitored by GC using *n*-tetradecane as internal standard. Products were silylated in GC vials with *N*-methyl-*N*-(trimethylsilyl)trifluoroacetamide. Response factors of the products with regard to *n*-tetradecane were obtained experimentally by analyzing known quantities of the substances. GC analyses were carried out using an HP-5 capillary column (phenyl methyl siloxane, 30 m × 320 × 0.25, 100/2.3-30-300/3) and a time program beginning with 2 min at 60 °C, heating rate 30 °C/min, 3 min at 300 °C. NMR spectra were measured at ambient temperature using CDCl_3_ as solvent, with proton, and carbon resonances at 300 MHz/400 MHz and 75 MHz, respectively. All NMR data are reported in ppm relative to the solvent signal. CHN-elemental analyses were performed with a Hanau Elemental Analyzer vario Micro cube.

Commercial substrates were used as received unless otherwise stated. All solvents and liquid reactants were degassed with Argon for 15 min prior to use. Ethylene was purchased from Air Liquide GmbH (purity 99,95%). All catalysts were donated by Umicore.

#### Preparation of CNSL

Cashew nutshell liquid was extracted following the procedure described in the reference [34]: Cashew nutshells (500 g), collected from Naliendele in Mtwara, Tanzania, were comminuted into ≈1 mm small particles which were than treated by Soxhlet extraction with Et_2_O (500 mL) at 50 °C for 6 h. Removal of the solvent in vacuo resulted in a highly viscous brown oil (160 g, 32 wt %). The CNSL was used without further purification.

#### Synthesis of 2-hydroxy-6-(non-8-enyl)benzoic acid (**2**) via ethenolysis of CNSL

A 1 L Parr autoclave was charged with the metathesis catalyst **Ru-1** (330 mg, 0.55 mmol), CNSL (37.7 g, 110 mmol) and DCM (100 mL) under ethylene atmosphere. The system was evacuated and backfilled with ethylene (5 bar) three times and finally pressurized to 10 bar. The mixture was stirred at 500 rpm at room temperature for 12 h. After the reaction time, the reaction mixture was filtered through celite and the filter cake was washed with DCM (2 × 10 mL). The solvent was removed in vacuo and the residue was dissolved in pentane (50 mL) and stored in the freezer until precipitation of the solid. The precipitate was filtered and washed with cold pentane (2 × 20 mL) yielding the product 2-hydroxy-6-(non-8-enyl)benzoic acid (**2**) as colorless solid (16,2 g, 84%). CHN-elemental analysis calcd for C_16_H_22_O_3_: C, 73.25; H, 8.45; found: C, 73.55; H, 8.53; ^1^H NMR (300 MHz, CDCl_3_) δ 10.98 (br. s., 1H), 7.38 (dd, *J* = 8.4, 7.5 Hz, 1H), 6.89 (dd, *J* = 8.3, 1.3 Hz, 1H), 6.79 (dd, *J* = 7.5, 1.3 Hz, 1H), 5.82 (ddt, *J* = 17.0, 10.2, 6.7, 6.7 Hz, 1H), 5.03 (q, *J* = 1.7 Hz, 1H), 4.89–4.99 (m, 1H), 2.94–3.05 (m, 2H), 2.00–2.10 (m, 2H), 1.56–1.68 (m, 2H), 1.29–1.44 (m, 8H) ppm; ^13^C NMR (75 MHz, CDCl_3_) δ 176.1, 163.7, 147.8, 139.2, 135.5, 122.8, 115.9, 114.1, 110.3, 36.4, 33.8, 31.9, 29.7, 29.3, 29.1, 28.9 ppm. The analytical data matched those reported in the literature [38].

#### Optimization of the reaction conditions for the synthesis of 2-hydroxy-6-(tridec-8-enyl)benzoic acid (**5**)

An oven-dried 20 mL vial was charged with **Ru-1** (3 mg, 5.00 μmol), **2** (131 mg, 0.5 mmol) and closed with a crimp cap. The vial was evacuated and backfilled three times with argon. 1-Hexene (3.50 mmol, 0.45 mL) and DCM (1 mL) were added simultaneously via syringe under an argon atmosphere. The continuous elimination of formed ethylene was performed by connecting the reaction vessel via an open system to an oil bubbler. The resulting mixture was stirred at 60 °C for 6 h. After the reaction was complete, the mixture was filtered through celite and the filter cake was washed with DCM (2 × 5 mL). The solvent was removed in vacuo and the residue was dissolved in pentane (5 mL) and stored in the freezer until precipitation of the solid. Product **5** was isolated as colorless solid (120 mg, 72%). CHN-elemental analysis calcd for C_20_H_30_O_3_: C, 75.43; H, 9.50; found: C, 75.43; H, 9.36; ^1^H NMR (400 MHz, CDCl_3_) δ 11.00 (s, 1H), 7.38 (t, *J* = 7.9 Hz, 1H), 6.86–6.91 (m, 1H), 6.76–6.82 (m, 1H), 5.33–5.44 (m, 2H), 2.95–3.03 (m, 2H), 1.92–2.08 (m, 4H), 1.56–1.66 (m, 2H), 1.25–1.43 (m, 12H), 0.86–0.92 (m, 3H) ppm; ^13^C NMR (75 MHz, CDCl_3_) δ 175.9, 163.7, 147.8, 135.5, 130.4, 130.3, 129.9, 129.8, 122.8, 115.9, 110.3, 36.5, 32.6, 32.3, 32, 31.8, 29.8, 29.6, 29.3, 29.1, 26.9, 22.3, 22.2, 14 ppm. The analytical data matched those reported in the literature [39].

#### One-pot synthesis of 2-hydroxy-6-tridecylbenzoic acid (**3**)

An oven-dried 20 mL vial was charged with **Ru-1** (3 mg, 5.00 μmol), **2** (131 mg, 0.50 mmol) and closed with a crimp cap. The vial was evacuated and backfilled three times with argon. 1-Hexene (3.50 mmol, 0.45 mL) and DCM (1 mL) were added simultaneously via syringe under an argon atmosphere. The continuous elimination of formed ethylene was performed by connecting the reaction vessel via an open system to an oil bubbler. The resulting mixture was stirred at 60 °C for 6 h. After the reaction was complete, methanol (0.5 mL) and activated charcoal (20.0 mg) were added. The vial was closed with a septum cap, penetrated with a cannula for pressure equilibration and placed into an autoclave. The system was purged twice with H_2_ (5 bar) and finally pressurized to 5 bar. The resulting mixture was stirred for 3 h at 50 °C. After cooling down to room temperature, the pressure was slowly released under constant stirring at 300 rpm. The reaction mixture was filtered through celite and the filter cake was washed with DCM (2 × 5 mL). The solvent was removed in vacuo and the residue was dissolved in pentane (5 mL) and stored in the freezer until precipitation of the solid. The precipitate was filtered and washed with cold pentane (2 × 5 mL), yielding the product **3** as colorless solid (120 mg, 72%). CHN-elemental analysis calcd for C_20_H_32_O_3_: C, 74.9; H, 10.1; found: C, 74.8; H, 9.8; ^1^H NMR (300 MHz, CDCl_3_) δ 10.98 (s, 1H), 7.38 (dd, *J* = 8.3, 7.6 Hz, 1H), 6.89 (dd, *J* = 8.3, 1.2 Hz, 1H), 6.79 (dd, *J* = 7.5, 1.1 Hz, 1H), 2.92–3.06 (m, 2H), 1.54–1.70 (m, 2H), 1.21–1.44 (m, 20H), 0.84–0.93 (m, 3H) ppm; ^13^C NMR (75 MHz, CDCl_3_) δ 176.1, 163.6, 147.9, 135.5, 130.3, 122.8, 115.9, 110.4, 36.5, 32.0, 31.9, 29.8, 29.69, 29.68, 29.65, 29.6, 29.5, 29.4, 29.3, 22.7, 22.2, 14.1 ppm. The analytical data matched those reported in the literature [40].

## Supporting Information

File 1 Additional screening and NMR spectra.
