# Supplementary material for: Synthesis of a tyrosinase inhibitor by consecutive ethenolysis and cross-metathesis of crude cashew nutshell liquid
Source: Beilstein J Org Chem. 2018 Oct 31;14:2737–44. doi: 10.3762/bjoc.14.252 (PMC6244364; doi:10.3762/bjoc.14.252)

Supporting Information

for

Synthesis of a tyrosinase inhibitor by consecutive ethenolysis and cross-metathesis of crude cashew nutshell liquid

Jacqueline Pollini, Valentina Bragoni, Lukas J. Gooßen*

Address: Lehrstuhl für Organische Chemie I, Ruhr-Universität Bochum, ZEMOS, Universitätsstraße 150, 44801 Bochum, Germany

Email: Lukas J. Gooßen* - lukas.goossen@ruhr-uni-bochum.de

*Corresponding author

Additional screening and NMR spectra

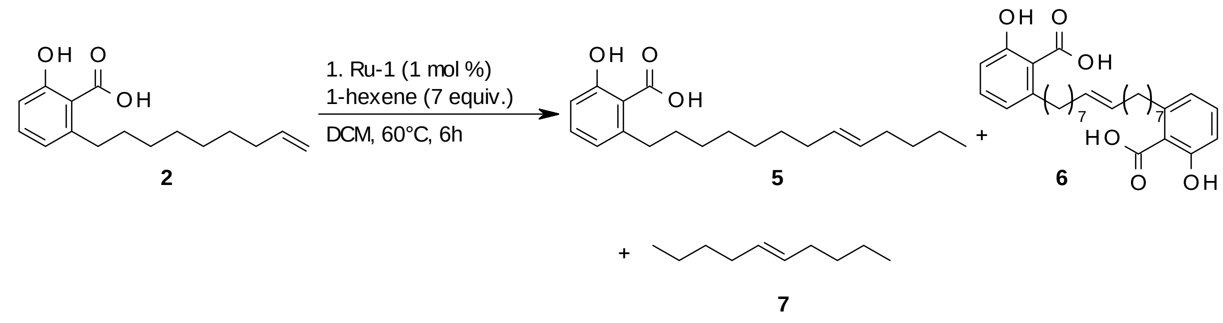

Scheme 1: Complete reaction equation of the hexenolysis step.

Additional screening of the reaction conditions

Table S1: Screening of the cross-metathesis of 2-hydroxy-6-(non-8-enyl)benzoic acid (**2**).

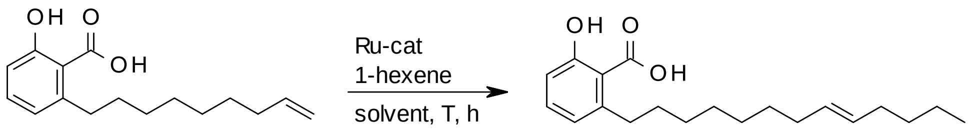

2 **5**

Entry	Solvent	1-hexene (equiv)	time	Ru-cat	conversion (%)	**5** (%)
1[a]	DCM	7	12 h	HG-1	65	33
2[b]	"	"	"	"	45	53
3	*p*-cymene	"	"	"	72	3
4	DEC	"	"	"	41	43
5	DMC	"	"	"	34	44
6	Et_2O	"	"	"	13	70
7	Me-THF	"	"	"	36	47
8	acetone	"	"	"	24	59
9	water	"	6 h	"	98	n.d.
10	toluene	"	"	"	41	51
11	1-hexene	"	"	"	33	61
12	THF	"	"	"	49	42
13	DCM	"	"	"	3	74
14[c]	"	"	"	"	2	72
15[d]	"	"	"	"	3	59
16	"	"	1 h	"	10	67
17	"	"	12 h	"	"	73
18[e]	"	"	"	"	3	76
19	"	"	6 h	M20	2	72
20	"	7	"	"	1	68
21	"	"	"	M2	2	65
22	"	7	"	"	1	65
23	"		"	M51	n.d.	56
24	"	"	"	M73 SiMES	1	54
25	"	"	"	M23	1	28
26	"	"	"	M31	7	55
27	"	"	"	G1	54	27
28	"	"	"	M22	29	2
29	"	"	"	HG-2	2	45
30	"	5	"	HG-1	6	69
31	"	3	"	"	19	65
32	"	10	12	"	0	73

Reaction conditions: 0.50 mmol 2, given equiv. 1-hexene, 1 mol% **Ru-cat**, 1 mL given solvent, given **T** (°C), given time, open system *via* oil bubbler; [a] room-temperature, closed system; [b] room-temperature; [c] 2 mL of solvent; [d] 0.5 mL of solvent; [e] 2 mol% Ru-**cat**. Yields were determined by GC using *n*-tetradecane as internal standard.

NMR spectra

2-Hydroxy-6-(non-8-enyl)benzoic acid (**2**, CAS 1629257-80-2)

^{1}H NMR (300 MHz, CDCl$_3$)

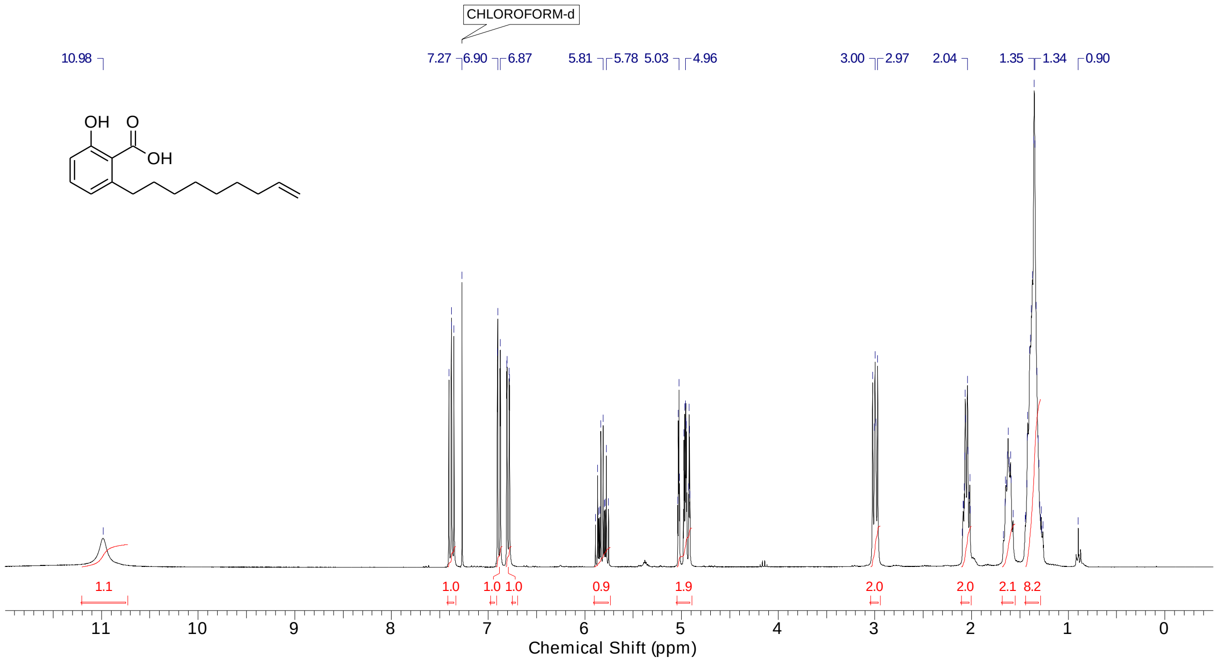

^{13}C NMR (75 MHz, CDCl$_3$)

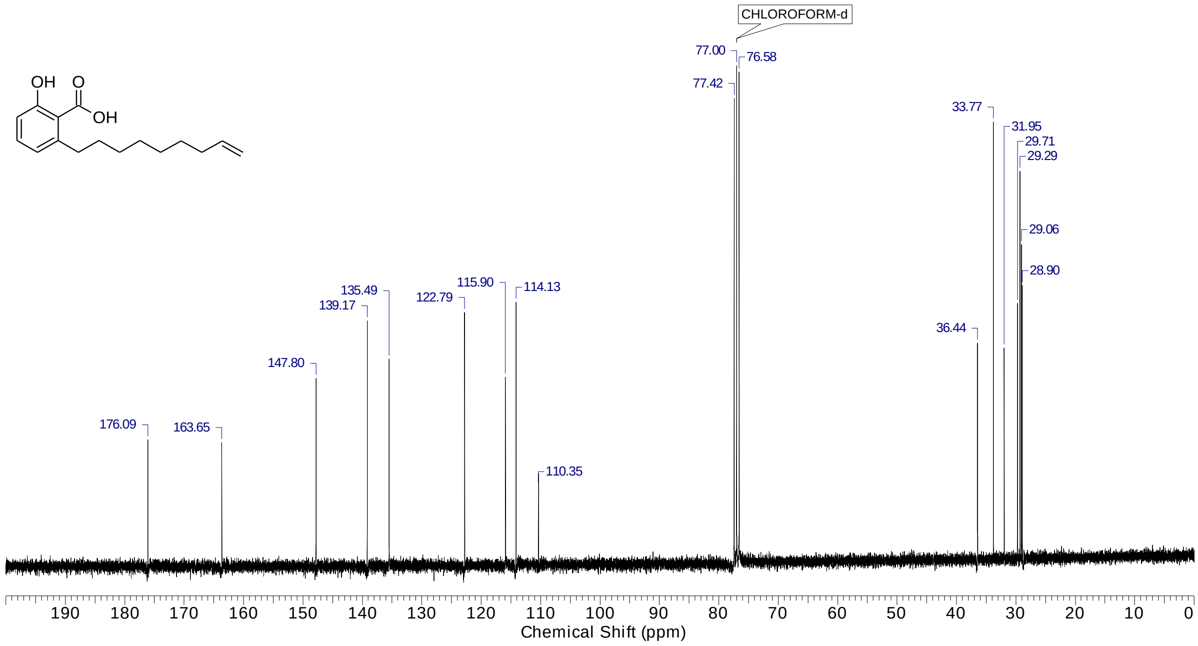

2-Hydroxy-6-(tridec-8-enyl)benzoic acid (**5**, CAS 88640-88-4)

^{1}H NMR (400 MHz, $CDCl_3$)

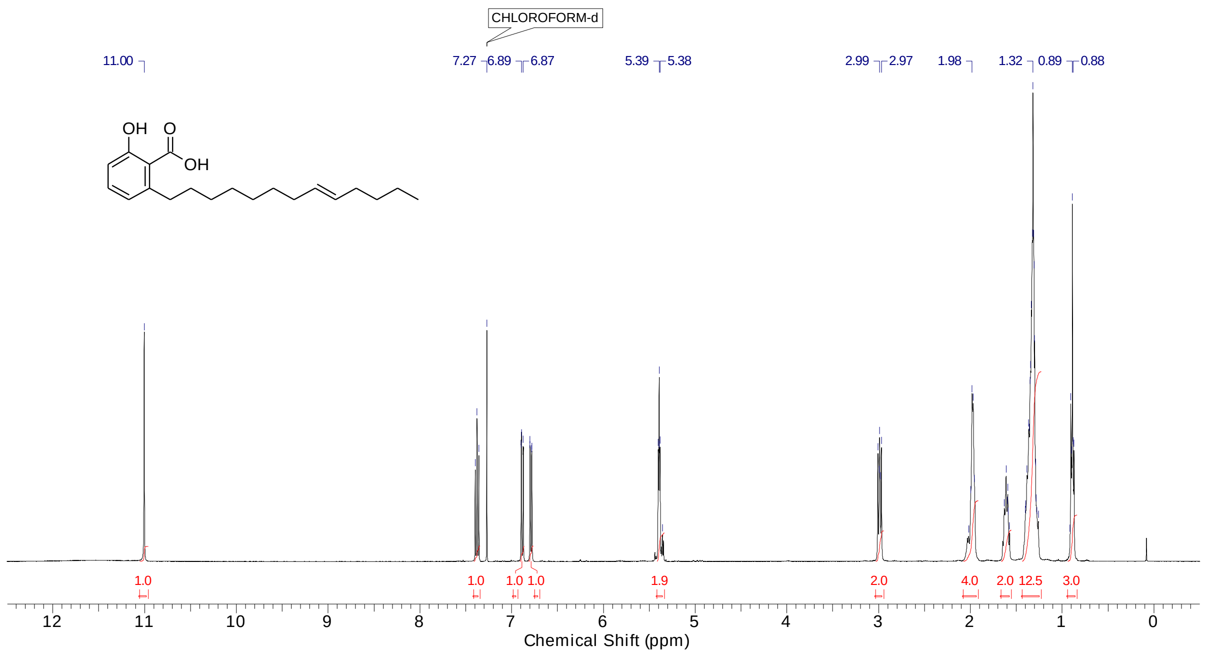

^{13}C NMR (75 MHz, $CDCl_3$)

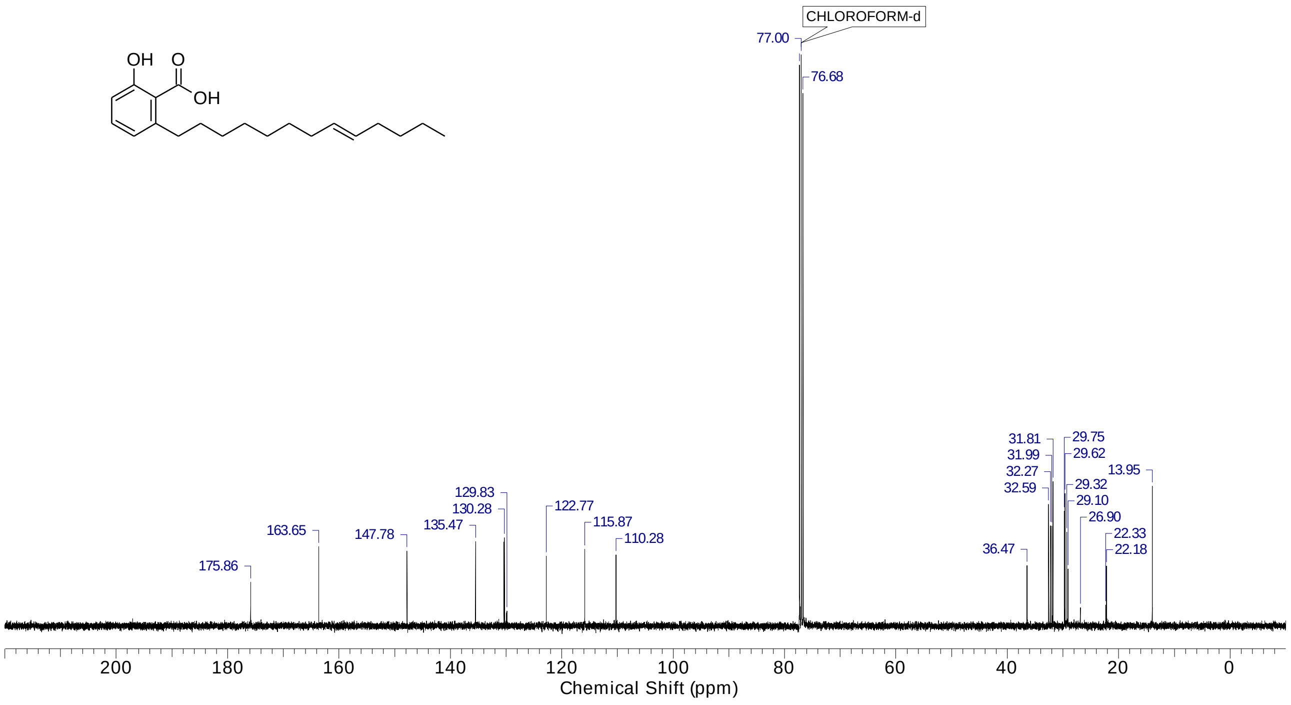

2-Hydroxy-6-tridecylbenzoic acid (**3**, CAS 20261-38-5**)**

^{1}H NMR (300 MHz, $CDCl_3$)

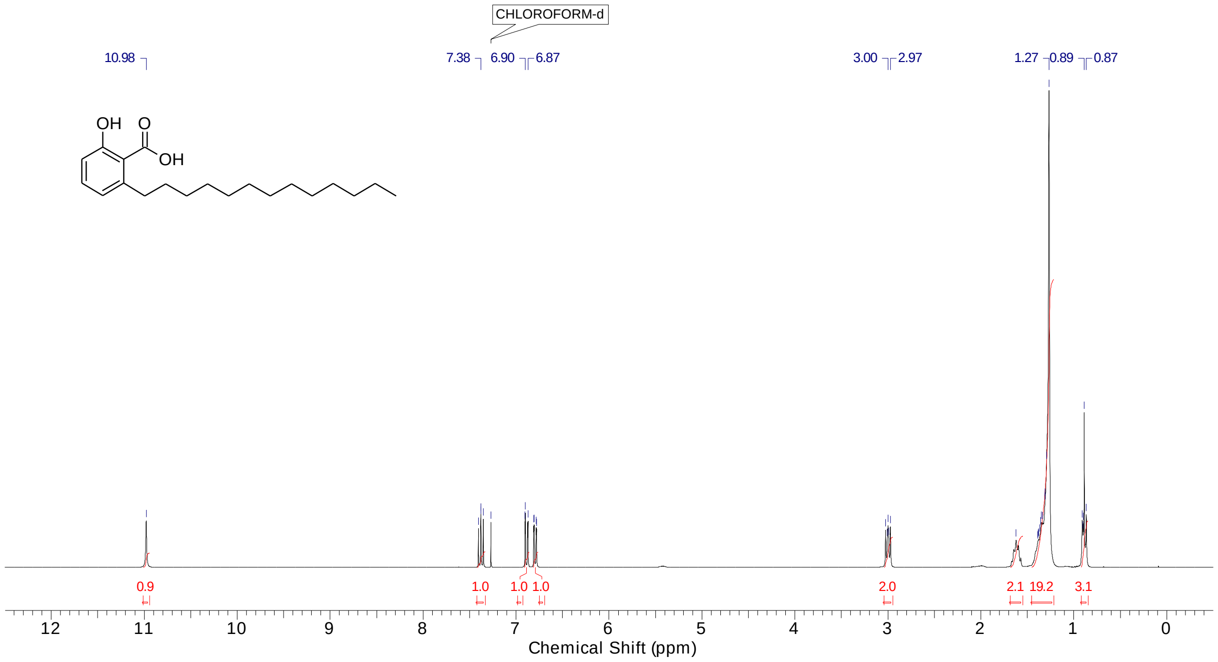

^{13}C NMR (75 MHz, $CDCl_3$)

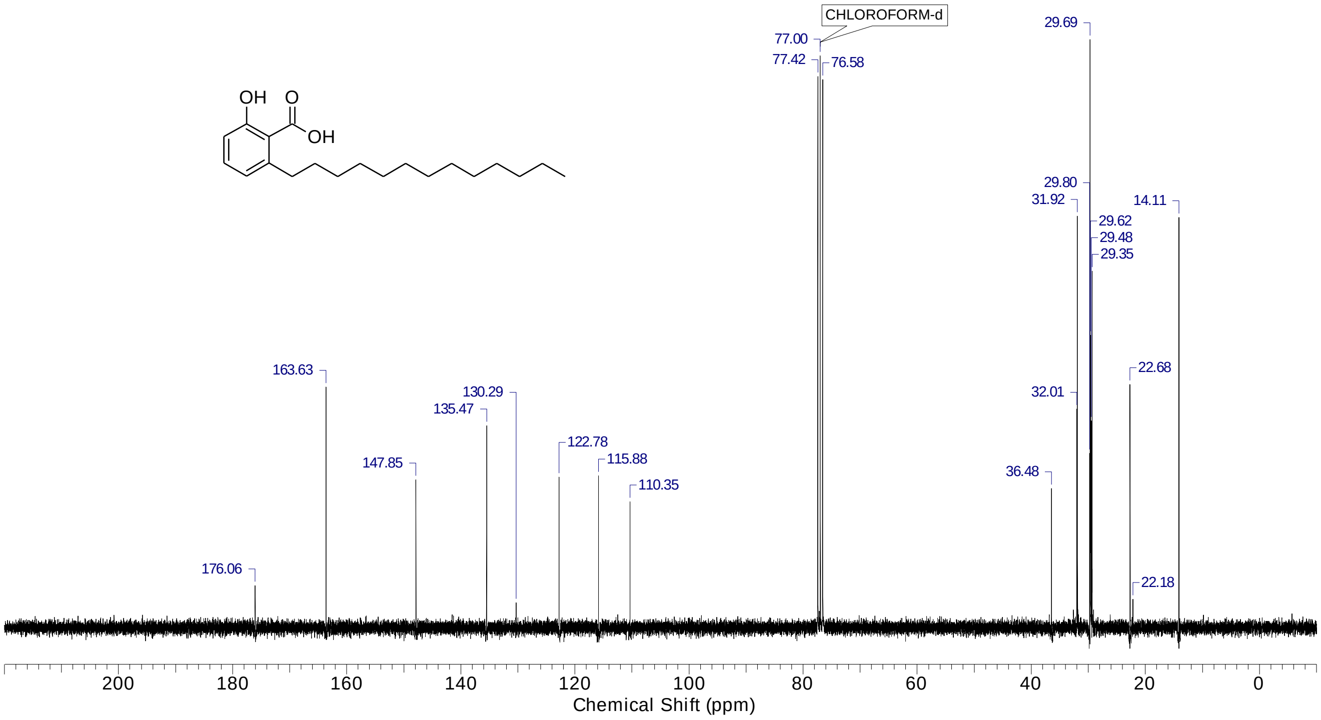

Supplement: File 1 — Additional screening and NMR spectra. [file Beilstein_J_Org_Chem-14-2737-s001.pdf]
